# Spheroid-induced heterogeneity and plasticity of uveal melanoma cells

**DOI:** 10.1007/s13402-022-00671-y

**Published:** 2022-04-11

**Authors:** Yao Chen, Xiaoqin Lu, Ling Gao, Douglas C. Dean, Yongqing Liu

**Affiliations:** 1 Hunan Key Laboratory of Ophthalmology, Eye Center of Xiangya Hospital, Central South University, National Clinical Medical Center for Geriatric Diseases of Xiangya Hospital, Changsha, China; 2 Department of Medicine, James Graham Brown Cancer Center, Birth Defects Center, University of Louisville School of Medicine, Louisville, KY USA; 3 Department of Ophthalmology, Second Xiangya Hospital, Central South University, Changsha, China

**Keywords:** Uveal melanoma, ZEB1, Cancer stem cell, Spheroid, Vasculogenic mimicry

## Abstract

**Purpose:**

The mechanism underlying cancer heterogeneity and plasticity remains elusive, in spite of the fact that multiple hypotheses have been put forward. We intended to clarify this heterogeneity in uveal melanoma (UM) by looking for evidence of cancer stem cell involvement and a potential role of ZEB1 in cancer cell plasticity.

**Methods:**

Spheroids derived from human UM cells as well as xenograft tumors in nude mice were dissected for signs of heterogeneity and plasticity. Two human UM cell lines were studied: the epithelioid type C918 cell line and the spindle type OCM1 cell line. We knocked down ZEB1 in both cell lines to investigate its involvement in the regulation of stem-like cell formation and vascularization by qRT-PCR, immunohistochemistry, flow cytometry, electrophoretic mobility shift assay (EMSA) and chromatin immunoprecipitation (ChIP) assays.

**Results:**

We found that a small side population (SP) in OCM1 showed stem cell-like properties such as heterogeneity, remote dissemination and nuclear dye exclusion after spheroid formation in vitro. ZEB1 regulated UM stem cell generation indirectly by promoting cell proliferation to form large size tumors in vivo and spheroid in vitro, and directly by binding to stemness genes such as *TERT* and *ABCB1*. In addition, we found that ZEB1 participates in vasculogenic mimicry system formation through the regulation of *CD34* and *VE-cadherin* expression.

**Conclusions:**

From our data we conclude that cancer stem cells may contribute to UM heterogeneity and plasticity and that ZEB1 may play a regulatory role in it.

**Supplementary Information:**

The online version contains supplementary material available at 10.1007/s13402-022-00671-y.

## Introduction

Uveal melanoma (UM) is the most common intraocular malignant tumor in adults, with a 10-year mortality rate of approximately 40%. The survival time decreases to less than 1 year once metastases are encountered, mostly in the liver, which occurs in almost half of the UM patients. Current treatment modalities for UM, such as brachytherapy, charged-particle radiotherapy, proton beam therapy, photodynamic therapy and surgical excision, are not beneficial to metastasis and overall survival [1, 2]. Besides, drug resistance limits the use of general chemotherapy for UM patients.

Histopathologically, UMs are classified into spindle, epithelioid and mixed cell types based on their cellular morphology [3]. A larger percentage of spindle cells in the tumor denotes a better survival, whereas the epithelioid phenotype is related to metastasis and a poor prognosis [4]. UM cells originate from neural crest-derived uveal melanocytes, which are initially of spindle morphology. How they turn into the epithelioid phenotype, what mechanism(s) underlies the phenotypic switch and their relation to malignancy are not clear. While the cellular heterogeneity of UMs is thought to be attributed to their neural crest origin [5], a possible subpopulation of UM cells displaying stem cell properties, featured by self-renewal and multidirectional differentiation, rendering tumor metastasis, drug resistance and recurrence, has been considered to be the cause [6, 7]. Differentiation of cancer stem cells may explain the different types of cancer cells in UM [8]. However, how these cancer stem cells are generated remains elusive. Transformed cells have lost their cell–cell contact inhibition property and they can overgrow in culture to form 3D spheroids that seem to mimic tumor formation in vivo [9]. The questions of whether and how tumor stem cells can be generated in such tumor cell spheroids are also not clear.

Zinc finger E-box binding homeobox 1 (ZEB1) is a transcription factor that promotes tumor invasion and metastasis by inducing epithelial-mesenchymal-transition (EMT) in carcinomas [10]. EMT not only plays an important role in embryonic development and malignant progression, but is also implicated in cancer therapy resistance [11] and contributes to cancer stem cells [12]. Recent studies have shown that cancer stem cells (CSCs) may originate from non-cancer stem cells and that ZEB1 plays a pivotal role in this process, as knockdown of ZEB1 could revert the CSC properties of prostate cancer cells such as colony-forming ability and the expression of canonical CSC markers (SOX2, CD44 and CD133) [13]. In addition, it has been found that exogenous ZEB1 expression can induce the acquisition of stem cell characteristics in breast cancer through the ZEB1/Ngn3 axis [14] and that high ZEB1 expression predicts radiotherapy relapse in triple-negative breast cancer, which is usually enriched in CSCs [15]. Moreover, ZEB1 overexpression has been found to lead to dedifferentiation and vascular invasion in cholangiocarcinoma, which promotes metastasis and stem cell features [16]. EMT is essential in cancer development and CSCs and Zeb1 have been implicated in the expression of several stem cell-associated transcription factors, including those with oncogenic potentials, such as BMI1, KLF4 and SOX2 [17, 18]. So far, EMT has been extensively studied in epithelial cancers such as breast [19] and lung [20] cancers. However, a role of EMT in mesenchymal tumors such as UM is still ambiguous. Previously, we reported that epithelioid UM cells exhibit a high ZEB1 expression, whereas spindle-type tumor cells show the opposite [21]. ZEB1 binds and represses the cyclin-dependent kinase inhibitor *CDKN1A* and the differentiation regulator *ID2*, but transactivates extracellular matrix degradation enzymes such as *MMP11* to induce UM cell dedifferentiation, proliferation and invasion. Nude mouse xenografts suggest that ZEB1 increases tumor growth and metastasis in vivo [21]. Hence, we speculate that ZEB1 may play a role in UM stem cell generation.

Herein we investigated the cellular heterogeneity of UM and found that a subpopulation in OCM1, a spindle type UM cell line, can develop heterogeneous cell types and show stem cell features through spheroid formation, which is related to ZEB1 activity. In addition, we found that the plasticity of UM cells featured as vascular mimicry, a flexible phenotype of tumor cells capable of recapitulating vascular channels without endothelial cells and pericytes [22, 23] and expressing CD34 [24] and VE-cadherin [25], can be promoted by ZEB1. Our data indicate that ZEB1 may play a key role in UM cell heterogeneity and plasticity. ZEB1 may serve as a prognostic UM biomarker, and modulation of ZEB1 expression may be used to design novel UM treatment modalities.

## Materials and methods

### Tumor samples

This study was approved by the Institutional Review Board of the University of Louisville and adhered to the tenets of the Declaration of Helsinki. Primary UMs were collected at the time of enucleation. Written informed consent was obtained. Tumor samples were processed for the preparation of paraffin-embedded sections.

### Animals

All athymic nude mice with BABL/c background were purchased from the Jackson’s Laboratory. Breeding, husbandry and surgical procedures were according to the ARVO Statement for the Use of Animals in Ophthalmic and Vision Research and were approved by the University of Louisville Institutional Animal Care and Use Committee (IACUC #17202) on June 13, 2017 and by the China Central South University Xiangya Medical School Ethics Committee (# 201703213) on March 6, 2017.

### Cell culture, Matrigel 3D culture and non-adherent spheroid preparation

Two widely used UM cell lines, C918 and OCM1, were kindly provided by Dr. Klara Valyi-Nagy, University of Illinois Chicago and cultured in DMEM with 10% heat-inactivated fetal bovine serum. Authentication of these two UM cell lines was confirmed by short tandem repeat (STR) analysis [26]. Mycoplasma-free cell cultures were confirmed using mycoplasma-specific PCR. For Matrigel 3D culture, cell culture plates were covered with 0.2 mm polymerized Matrigel (BD Biosciences) before seeding UM cells. For non-adherent spheroid preparation, UM cells were scraped off the culture plates and pipetted up and down to obtain single cell suspensions and transferred to ultra-low adherent dishes to form spheroids of 500‒800 cells in suspension for 2–3 days. Thereafter, the spheroids were transferred to culture plates coated with the Matrigel (1:160 dilution) to let the spheroids settle down and let cells inside the spheroids migrate out.

### Flow cytometry analysis

OCM1 spheroid-derived cells were digested with 0.25% trypsin from culture plates, suspended in prewarmed DMEM, and stained with Hoechst 33,342 dye (Invitrogen, 1:500) for 90 min at 37 °C. Next, the cells were washed and resuspended in PBS, after which Hoechst dye negative side population (SP) cells were identified and isolated using a MoFlo cell sorter (Dako; Carpinteria, CA, USA) after excitation of the Hoechst dye with a 350 nm UV laser (100 mW power was used). Fluorescence light emitted by cells was directed toward a 510 nm DCLP dichroic mirror and collected simultaneously by two independent detectors following 450/65 nm and 670/30 nm bandpass filters, respectively. The flow cytometric data were analyzed using FlowJo.

### Immunofluorescence (IF) and immunohistochemistry (IHC)

Cells were cultured in 8 well chambers until confluence. Next, they were fixed with 4% paraformaldehyde for 15 min and washed with phosphate-buffered saline (PBS) for IF. Formalin-fixed and paraffin-embedded xenograft tumor sections were deparaffinized by sequential incubations with xylene, gradually decreased concentrations of ethanol and PBS, followed by an antigen retrieval process in a citrate buffer at 95℃ for 15 min before IHC. Blocking solution consisting of 4% serum of a specified animal used to raise the second antibody, 0.8% bovine serum albumin (BSA) and 0.1% Tween-20 in PBS were added for 1 h at 25℃. The samples were subsequently incubated overnight at 4℃ with primary antibodies in blocking solution. After the primary antibodies were removed and the samples washed, secondary antibodies were applied for 1 h at 25℃. The primary antibodies used were: mouse anti-CD34 (Thermo Scientific, 1:300) and rabbit anti-ZEB1 (Santa Cruz 1:100). Bound antibodies were visualized using an Alexa fluor-568 secondary antibody conjugated with red fluorescein (Invitrogen, 1:500). Nuclei were counterstained with Hoechst 33,342 dye (Invitrogen, 1:500) and images were captured using a Zeiss fluorescence microscope (Zeiss, Axiovert 200). For formalin-fixed and paraffin-embedded patient tumor sections, staining was visualized using a 3-Amino-9-ethylcarbazole kit (AEC; Zhongshan, China) and the sections were counterstained with 4′,6-diamidino-2-phenylindole (DAPI) (Zhongshan, China).

### Lentiviral knockdown of ZEB1

OCM1 and C918 cells were transfected with a validated lentiviral short-hairpin RNA (shRNA) mixture of 4 shRNA sequences directed against human *ZEB1* and 1 scramble fragment as a vector control, which was purchased from Genechem Technologies (Shanghai, China) as reported previously [21]. The target sequence (5’- AACAATACAAGAGGTTAAA -3’) was selected to downregulate ZEB1 in vitro [21] based on the result of a blast against the human genome – all known ZEB1 transcript variants contain this short sequence. Lentiviral particles were produced by a four-plasmid transfection system. Briefly, 293 T cells were transfected with the lentiviral vector and packaging plasmids, after which the supernatants containing recombinant pseudo-lentiviral particles were collected from culture dishes on the second and third day after transfection.

### RNA extraction and real-time quantitative PCR (qRT-PCR)

RNA was extracted using TRIzol solution (Invitrogen, Carlsbad, CA, USA). Complementary DNA (cDNA) was synthesized using an Invitrogen RT Kit according to the manufacturer’s protocol (Invitrogen). SYBR Green qPCR was performed using a Mx3000P Real-Time PCR System (Stratagene, Cedar Creek, TX, USA) according to the manufacturer’s instructions. Information about the PCR primers used is presented in Supplemental Table S1. Three independent samples, each in technical triplicate, were analyzed for each qRT-PCR condition, and right-sized amplicons were validated by agarose gel electrophoresis.

### Electrophoretic mobility shift assay (EMSA)

A detailed EMSA protocol has been described previously [27]. Briefly, C918 UM cells were lysed and subsequently served as non-denatured protein extract. Based on the human CD34 promoter sequence, the sequence 5′- CTCCATTCCAACTGGGAGGACC -3′ with a putative ZEB1-binding site (CAACTG) was selected for double strand oligo synthesis with and without an artificial mutation in the ZEB1 site (mutant oligo sequence: 5′- CATACCCCAAAATTTTAAACACAG -3′). For the human CD105 promoter, the sequence 5′- TTCCTCATCTGCAAAATGGGATCA -3′ with a putative ZEB1 site (CATCTG) was selected for double strand oligo synthesis with and without an artificial mutation in the ZEB1 site (mutant oligo sequence: 5′- TTCCTGCTGCACAAAATGGGATCA -3′). The crude protein lysate was mixed with the above oligos separately. Binding reactions without protein lysate or oligos served as negative controls. All reaction samples were electrophoresed on 6% native polyacrylamide gels and then visualized under UV light after being stained with SYBR green (Molecular Probe).

### Chromatin immunoprecipitation (ChIP) assay

ChIP assays were carried out as described previously[28] using formaldehyde to crosslink genomic DNA of C918 cells. The chromatin was sheared to an average length of 500 bps. An anti-ZEB1 antibody was used for immunoprecipitation and IgG was used as a negative control. Immunoprecipitation with a histone 3 antibody (H3) in EpiTect kit (Qiagen, cat. GAH-2206) was used as a positive control. Sequences of the primers used for the tested promoters and the expected sizes of the PCR products are shown in Supplemental Table S1.

### Intravitreal injection

Four 6 weeks old mice were anesthetized, after which OCM1 or C918 cells were delivered into the vitreous of the mice as described previously [21]. 30 days after implantation, the mice were euthanatized by CO_2_, and the eye and liver tissues were collected and fixed overnight in 10% neutral buffered formalin and then in 70% ethanol until further processing and embedding in paraffin. Sections of 10 µm were prepared for histopathological and immunostaining analyses.

### Statistical analyses

Student’s t-test or one-way ANOVA were conducted for two or three independent animal and cell samples, respectively, after F-test confirmation that the compared samples show equal levels of variance. All values in the graphs are presented as means ± standard deviations (SD). ‘***’ means *p*-value < 0.001, ‘**’ means *p*-value < 0.01, whereas ‘*’ means *p*-value < 0.05. For in vitro studies including qRT-PCR, results were obtained from at least 3 independent experiments of three technical replicates.

## Results

### UM heterogeneity in patients and model animals

**Fig. 1:**
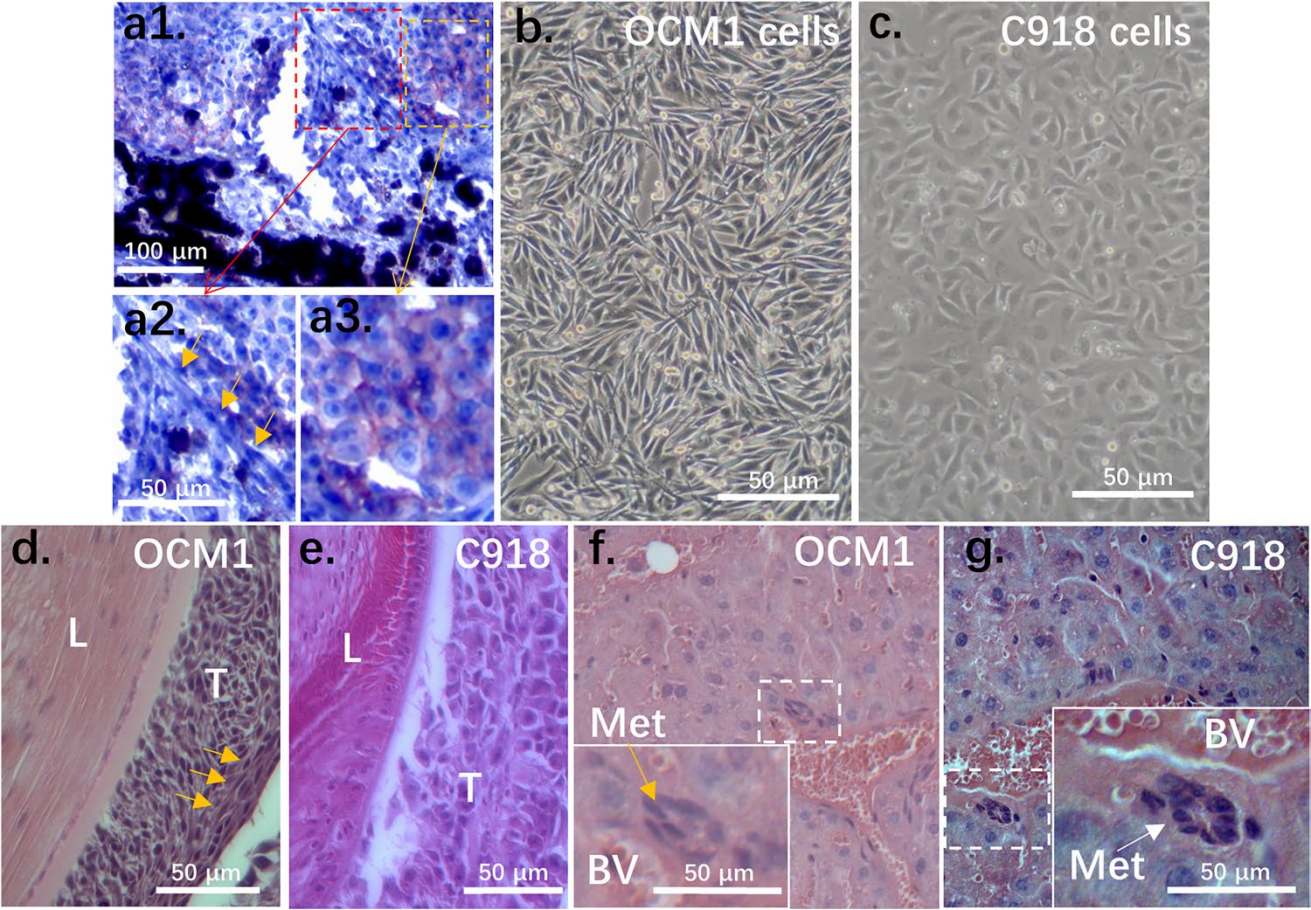
Morphology of a primary UM, UM cells, and their grafted and metastasized tumors. (**a1**) A representative primary UM that contains both (**a2**) spindle and (**a3**) epithelioid cell types. The epithelioid cells were stained with the epithelial marker E-cadherin. (**b**) The spindle type UM cell line OCM1 and (**c**) the epithelioid type UM cell line C918 were selected for analysis. Representative 30-day grafted tumors of both (**d**) OCM1 and (**e**) C918 cells are shown. Both grafted OCM1 (**f**) and C918 UM (**g**) cells could metastasize to the livers to form metastatic nodules (Met) nearby blood vessels that can be distinguished from local liver cells. L, lens; T, tumor; Yellow arrows point to the spindle type tumor cells while a white arrow points to epithelioid tumor cells; BV, blood vessel; Met, metastasized tumor

Uveal melanocytes have a spindle cell morphology. Melanocyte-derived UMs, however, exhibit both spindle and epithelioid cell morphologies [4]. Both cellular types may simultaneously be present at the same site of a tumor (Fig. 1a). To understand this heterogeneity, we injected cells of two representatives UM cell lines into the vitreous of nude mice (OCM1, spindle phenotype; C918, epithelioid phenotype) (Figs. 1b, c). The primary grafted tumors and liver metastatic lesions were investigated 1 month after intravitreal injection. We found that the spindle type OCM1 cells generated both spindle and epithelioid tumors (Fig. 1d), whereas the epithelioid C918 cells solely generated epithelioid tumors (Fig. 1e), indicating that the transformation from spindle to the epithelial cell type in UMs evolves in a one-way direction. Within a month, both C918 and OCM1 grafts metastasized to the livers, and the metastasized tumor cells were either of the epithelioid phenotype for C918 or the mixed spindle-epithelioid phenotype for OCM1, as expected (Figs. 1f, g), suggesting that both spindle and epithelioid tumor cells are invasive and metastatic. Based on these results, we hypothesize that the spindle OCM1 cell line represents less mature tumor cells that may possess cancer stem cell properties allowing them to generate various cell types, whereas the epithelioid C918 cell line represents more differentiated tumor cells that can only reproduce themselves.

### OCM1 cells can display stem cell-like properties after in vitro spheroid formation

**Fig. 2:**
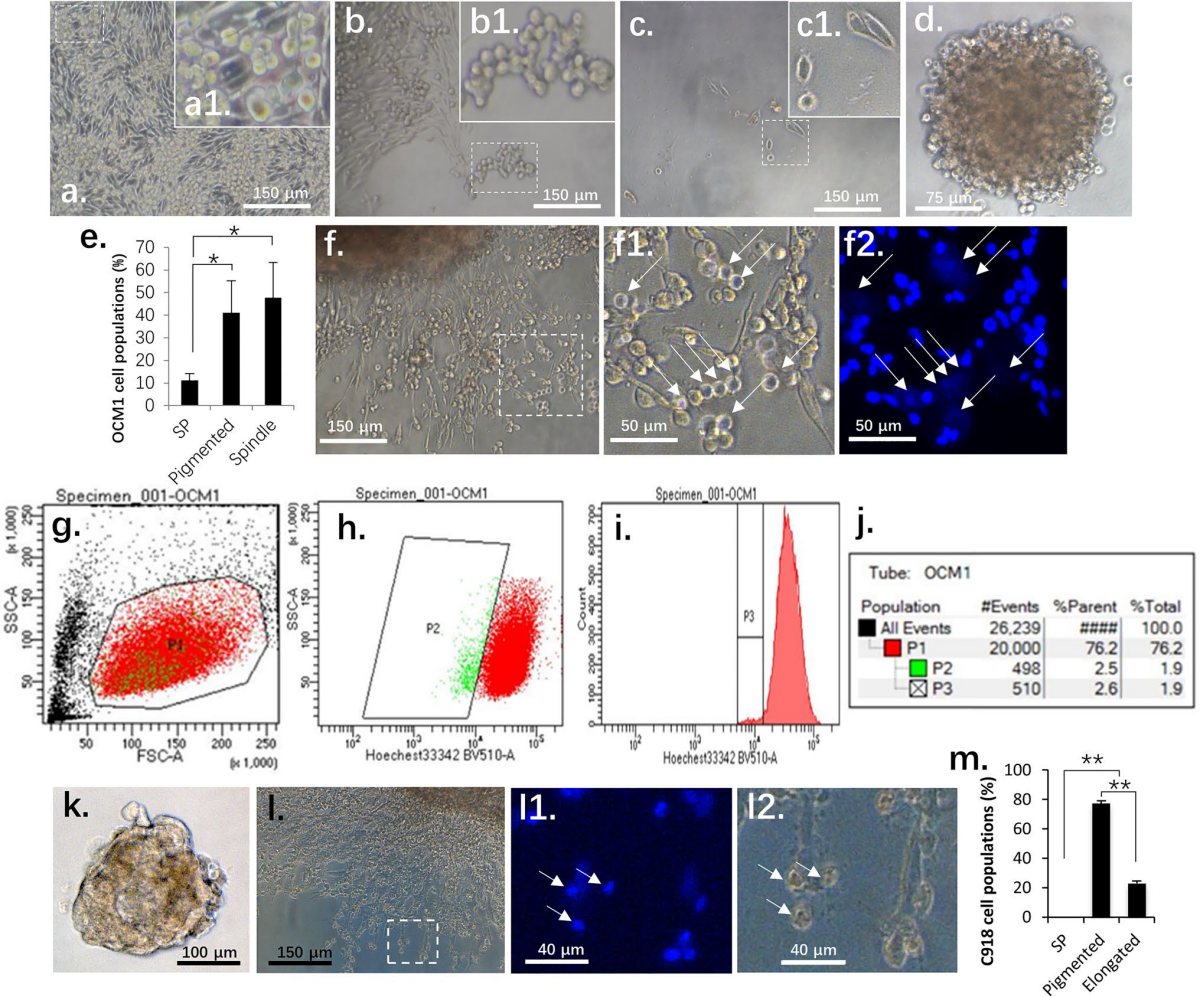
OCM1 cells exhibit stem-like cell properties after spheroid formation in vitro. (**a**) In addition to the original spindle type cells, OCM1 spheroids also generate pigmented epithelioid-like cells. (**b**) These epithelioid-like cells can survive suspension and (**c**) travel over long distances. (**d**) Single OCM1 cell-formed spheroid. (**e**) Percentages of the three major OCM1 spheroid-derived cell populations including cancer stem-like cells, also known as side population (SP). (**f**) A representative OCM1 spheroid and its derived cell populations. (**g**) A flow dot-plot and gate (P1) for similar sized cells. (**h**) Dot-plot gate (P2) for Hoechst dye negative cells. (**i**) Histography gate (P3) for Hoechst dye negative cells. (**j**) Hoechst dye negative cell rates. (**k**) Epithelioid C918 cells can form small compacted spheroids in vitro. (**l**) C918 spheroid-derived cells exhibit two phenotypes, with yellow epithelioid cells often sitting on the top of the other adherent elongated cells. (**m**) Percentages of the three major C918 spheroid-derived cell populations. White arrows point to Hoechst dye negative cancer stem-like cells. *, significant at *p* < 0.05; **, significant at *p* < 0.01

To clarify whether spindle-type OCM1 cells represent cancer stem-like cells or not, we cultured OCM1 cells in monolayer and stained them with nuclear Hoechst dye at 37℃ for 30 min. Cancer stem cells possess vast numbers of nuclear membrane transporters such as ABCG2 that can pump small chemicals like Hoechst dye out of the nuclei, thereby keeping them stain negative [29]. No such cancer stem cells were identified, since all monolayer cultured OCM1 cells were Hoechst positive similar to C918 cells (Supplemental Fig. S1). The notion that OCM1 may generate cancer stem-like cells in grafted tumors led us to postulate that the 3D structure of solid tumors may create a microenvironment allowing stemness gene reactivation in some of the cells and reprogram them into a stem-like status. To mimic grafted tumor formation, we cultured OCM1 spheroids on non-adherent plates (see Methods) for 3 days. After doing so, we observed a group of small and round pigmented cells (Fig. 2a) migrating out of the spheroids and congregating to form aggregates (Fig. 2b). These cells survived suspension culture and dispersed to remote areas, re-attached and transformed back to their initial spindle phenotype (Fig. 2c). The ability of OCM1 cells to survive suspension culture, remote migration and phenotypic transformation are alike the features of cancer stem cells. To confirm cancer stem cell properties and to determine the percentage of cells with such properties, spheroid-derived OCM1 cells were treated with 0.25% trypsin to obtain single cell suspensions and cultured in ultra-low adherent 96-well plates. Some of these OCM1 cells not only survived suspension culture, but also kept proliferating and conglomerated into spheroids after two weeks (Fig. 2d). About half of the spheroid-derived cells maintained their spindle phenotype, whereas the others became pigmented and epithelioid (Fig. 2e), which is consistent with the above observation that OCM1 graft-derived UMs encompass both spindle and epithelioid phenotypes (Fig. 1d). In addition, we found that about 10% of the epithelioid population was Hoechst dye negative and unpigmented (Figs. 2f, e). To validate the immunostaining data, we sorted single spheroid-derived OCM1 cells by flow cytometry after being stained with Hoechst dye, and found that about 1.9% of the sorted cells was Hoechst dye negative (Figs. 2g – j). Taken together, these data indicate that OCM1 spheroids can generate cancer stem-like cells that may promote tumor heterogeneity (Figs. 1a, d).

### Epithelioid C918 cells cannot generate Hoechst dye negative side populations (SP) after spheroid formation

**Fig. 3:**
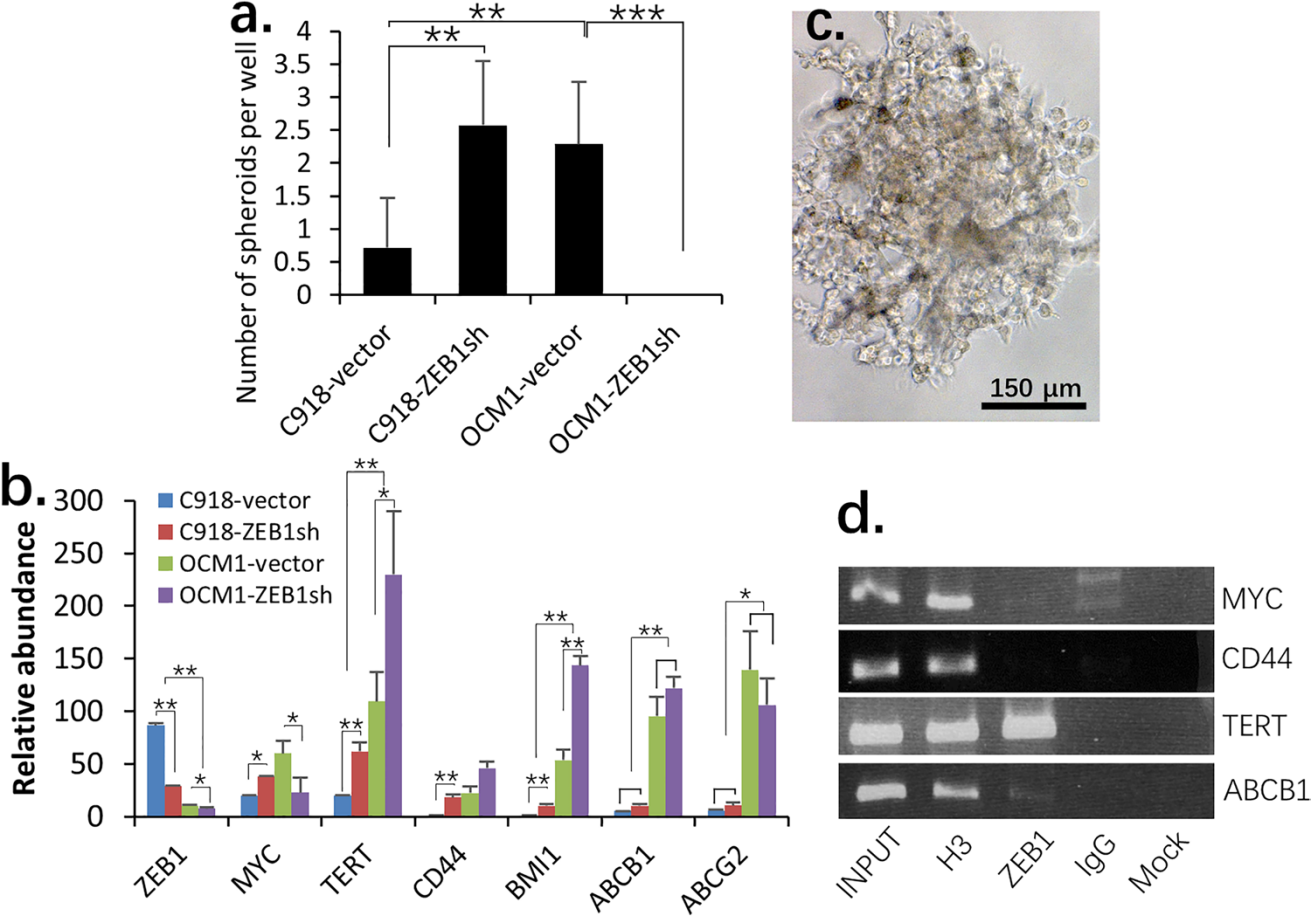
ZEB1 regulates stemness genes of UM cells. (**a**) Bar graph showing the capacities of C918-vector, C918-ZEB1sh and OCM1-vector spheroid derived cells to form spheroids in suspension and showing that knockdown of ZEB1 in OCM1 (OCM-ZEB1sh) cells completely diminishes their capacity to form spheroids in suspension. (**b**) Expression of stemness genes in C918-vector, C918-ZEB1sh, OCM1-vector and OCM1-ZEB1sh cells. (**c**) A representative C918-ZEB1sh cell spheroid. (**d**) ChIP assays showing that ZEB1 directly binds to and potentially represses expression of the TERT and ABCB1 genes. INPUT, 10% of the original chromatin before immunoprecipitation; H3, pan histone 3 antibodies as a positive control; IgG, isotype negative control; Mock, negative control without primary antibody; *, significant at *p* < 0.05 **; at *p* < 0.01; ***, at *p* < 0.001

To verify whether epithelioid C918 cells can, similar to spindle OCM1 cells, generate cancer stem-like side populations (SP), we cultured C918 spheroids in the same way as OCM1 spheroids (Fig. 2k). The C918 spheroids were smaller than the OCM1 spheroids and might have spheroid-derived cells that consist of two major populations — one with a yellow epithelioid morphology, positioned on the other adherent elongated branch-like cells (Fig. 2l) when placed back for adherent culture as with the OCM1 spheroids. By doing so, no Hoechst dye negative cells were identified among the C918 spheroid-derived cells (Figs. 2l, m). Similarly, we dispersed these spheroids-derived C918 cells into single cell suspensions and cultured them in ultra-low adherent 96-well plates. We found that over 2% of the OCM1 single-cell cultures could form spheroids while less than 1% of C918 single-cell cultures could do so (Fig. 3a). It appeared that the yellow epithelioid C918 cells were those that survived suspension and traveled long-distances similar to OCM1 cells (Fig. 1f). This result was consistent with those obtained with the above C918 graft tumors and metastasized tumors that only comprised epithelioid cells (Figs. 1e, g), suggesting that the epithelioid C918 cells are terminally differentiated and highly malignant cells that lack the flexibility of OCM1 cells to be reprogramed into cancer stem-like cells to become more heterogeneous after spheroid and tumor formation both in vitro and in vivo.

### ZEB1 represses the expression of UM cancer stemness genes

**Fig. 4:**
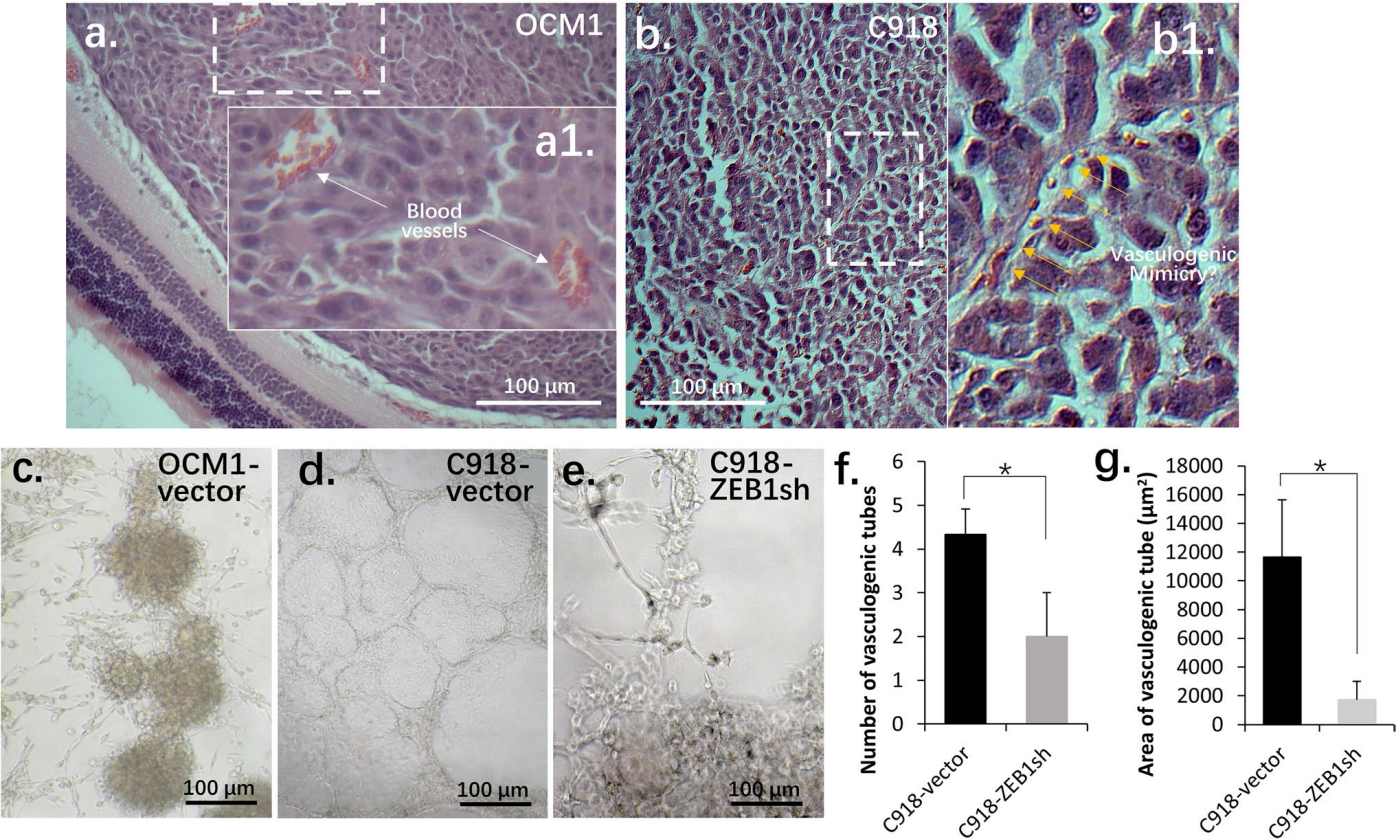
Blood vessels in UM cell-grafted tumors and vasculogenic mimicry in UM cell 3D cultures. (**a**) A representative section of a 30-day OCM1-grafted tumor showing that the tumor is confined to the vitreous and that several blood vessels are formed within the tumor. (**b**) A representative section of a 30-day C918-grafted tumor showing that the tumor has broken the vitreous and has invaded the surrounding tissue; some erythrocytes and leukocytes can be spotted within the tumor mass. (**c**) Matrigel 3D culture of OCM1-vector cells that piled up to form spheroids. (**d**) Matrigel 3D culture of C918-vector cells that formed vasculogenic mimicry structures while (**e**) ZEB1 knockdown in C918 (C918-ZEB1sh) significantly reduced (**f**) the number of vasculogenic tubes and (**g**) the average area of such tubes. White arrows point to blood vessels whereas yellow arrows point at potential vasculogenic mimicry; *, significant at *p* < 0.05

Previously we reported that ZEB1 may regulate multiple oncogenic components during UM progression [21]. To understand whether ZEB1 also takes part in spheroid-induced side population generation, we knocked down ZEB1 in OCM1 cells using lentiviral ZEB1 shRNA [21] and seeded single OCM1-ZEB1sh (ZEB1 knock-down) cells on ultra-low adherent 96 wells and cultured them for 2 weeks to form spheroids. We found, however, that OCM1-ZEB1sh did not survive in suspension and, thus, no spheroids were formed (Fig. 3a), indicating that decreased ZEB1 expression in OCM1 cells may block their spheroid-forming ability. We subsequently investigated whether ZEB1 affects cancer stemness genes in OCM1. Among the selected cancer stemness genes, we found that *TERT, MYC, CD44, BMI1, ABCB1* and *ABCG2* were highly expressed in OCM1 cells compared to their expression in C918 cells, whereas the expression of *ZEB1* was opposite to that of the stemness genes (Fig. 3b). We found that the number of spheroids formed from the single-cell suspension culture and the expression of the stemness genes were both negatively correlated with the ZEB1 expression level (Figs. 3a, b), suggesting that ZEB1 may suppress cancer stem cell properties in UM. To verify this suggestion, we knocked down ZEB1 in C918 [21] and found that ZEB1 down-regulation increased the expression of the above stemness genes in C918 (Fig. 3b). We also seeded and cultured these single C918-ZEB1sh cells on ultra-low adherent 96-well plates to form spheroids. As a result, we found that the C918-ZEB1sh cells-formed spheroids were relatively bigger (Fig. 3c) and 4 times more abundant than the original C918 cells (Fig. 3c), indicating that ZEB1 acts as a repressor of stemness genes in UM. As knockdown of ZEB1 in OCM1 led to OCM1-ZEB1sh cell death in suspension (Fig. 3a), we speculated that a minimum amount of ZEB1 was required for UM cells to survive in suspension. ZEB1 acts as a transcription factor by directly binding to E-box elements (consensus sequence, CANNTG), which are located within the promoter regions of target genes. We found such E-box elements in the putative promoter regions of the *MYC, TERT, CD44, ABCB1* and *ABCG2* genes, but not in the putative promoter region of the *BMI1* gene. To assess whether ZEB1 directly or indirectly regulates these stemness genes, we performed immunoprecipitation (ChIP) assays using an anti-ZEB1 antibody and crosslinked C918 chromatin. We found that ZEB1 could directly bind and inhibit the expression of both the *TERT* and *ABCB1* genes (Fig. 3d). The ChIP assays did not show that ZEB1 direct binds to the promoter regions of *MYC* and *CD44*, so it may indirectly regulate them. ChIP-PCR failed to amplify the promoter of the *ABCG2* gene, likely due to its high GC content.

### C918 cells show vasculogenic mimicry in 3D culture

Tumor progression is strongly correlated with blood supply. UM is a blood vessel enriched tumor and metastasizes through the circulation system [30]. Previously, we showed that in C918 cells ZEB1 is highly expressed while OCM1 cells exhibit a relatively low ZEB1 expression (Fig. 3b) [21]. As a result, C918 grafted tumors grew faster and were more invasive than OCM1-grafted tumors (Figs. 4a, b) [21]. Within 30 days, the C918 grafted tumors had completely broken the vitreous and had metastasized to the liver (Fig. 1g) while the OCM1-grafted tumors were still confined to the vitreous without invasion into their surrounding ocular tissues, although they did metastasize to the liver (Fig. 1f). Unlike OCM1-grafted tumors where several blood vessels were distributed throughout the tumor masses (Fig. 4a), no normal blood vessel was formed in C918-grafted tumors, although some erythrocytes and leukocytes were spotted in the tumors (Fig. 4b). This observation suggests that blood cells could invade into the tumor tissue through an unidentified route. Vasculogenic mimicry, vessel tubes formed by tumor cells instead of endothelial cells, does occur in UM and is regarded as a sign of cancer cell plasticity (Fig. 4b) [31]. To clarify whether C918 cells can form such vasculogenic mimicry, we performed Matrigel 3D cultures and found that these epithelioid cells could indeed form vessel-like tubes whereas the spindle OCM1 cells only generated spheroid-like pileups (Figs. 4c, d), indicating that the blood cells in the C918-grafted tumors were likely delivered by this vasculogenic mimicry system. In addition, we found that ZEB1 knockdown in OCM1 led to cell death in suspension or Matrigel culture while ZEB1 knockdown in C918 reduced the numbers and areas of the vasculogenic tubes formed in Matrigel culture (Figs. 4e - g), suggesting that ZEB1 may positively regulate vasculogenic mimicry formation of C918 cells.

### ZEB1 participates in vascular mimicry formation of UM cells

**Fig. 5:**
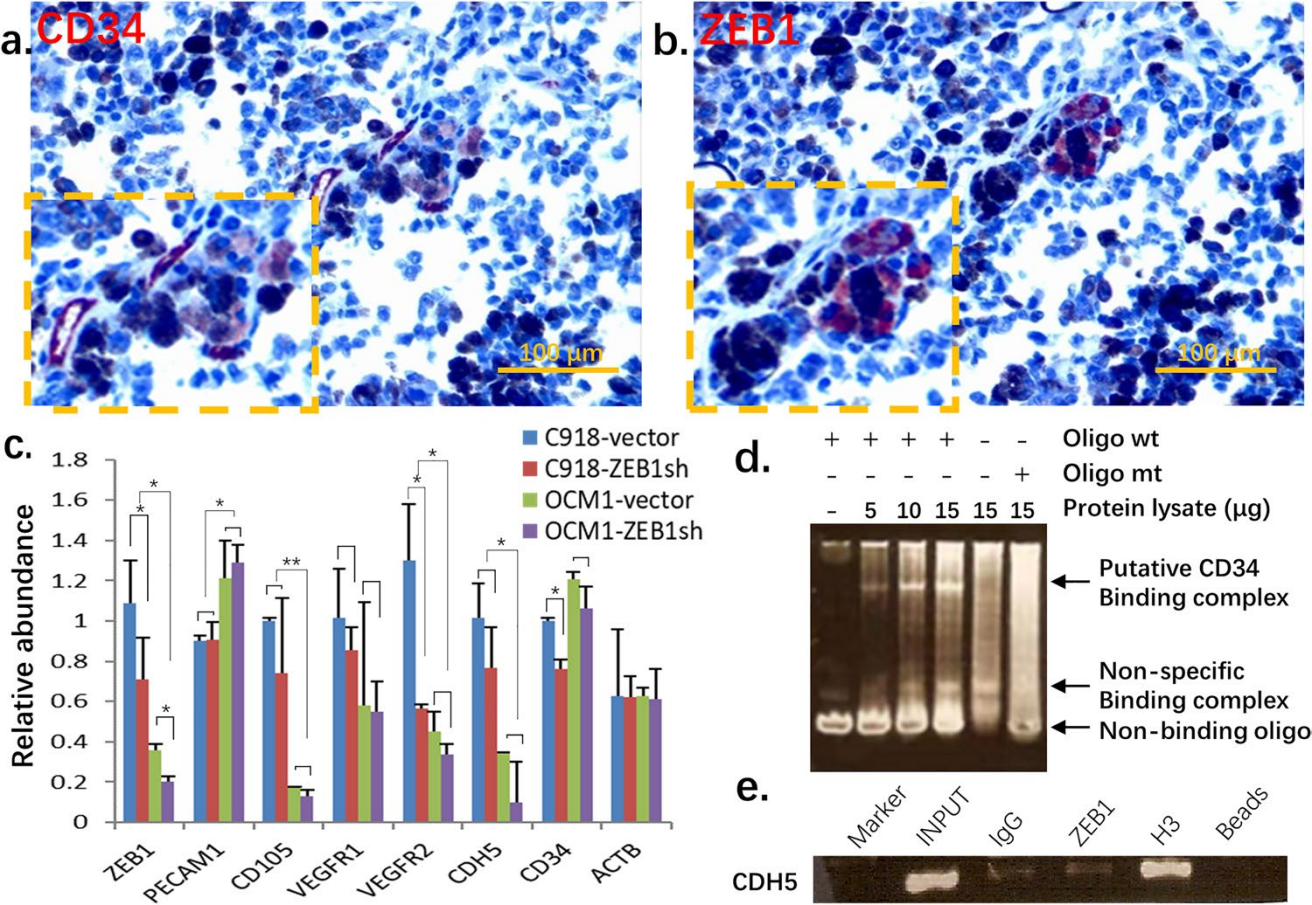
ZEB1 regulates genes involved in UM vascularization. (**a**) The endothelial precursor marker CD34 colocalizes with (**b**) ZEB1 in consecutive sections. (**c**) The expression level of ZEB1 positively correlates with that of genes involved in tissue vascularization. (**d**) Electrophoretic mobility shift assay (EMSA) of ZEB1 binding to the *CD34* promoter confirms that ZEB1 binds to and likely regulates its expression. (**e**) Chromatin immunoprecipitation (ChIP) assay confirming that ZEB1 binds to *CDH5* and likely regulates its expression. wt, wild type; mt, mutant

CD34, an endothelial precursor marker, is indicative of vascularization of UM. We observed co-expression of both ZEB1 and CD34 in human UM adjacent sections (Figs. 5a, b), implying that ZEB1 activation might be associated with CD34 expression. Indeed, we found that ZEB1 downregulation in C918 and OCM1 led to a decrease in CD34 expression (Fig. 5c). An electrophoretic mobility shift assay (EMSA) showed that a putative ZEB binding complex was formed when a C918 protein lysate was mixed with a wildtype (wt) ZEB1 binding oligo specific for the promoter sequence of the *CD34* gene, whereas this putative ZEB1 binding complex decreased when the protein lysate was mixed with a mutated (mt) oligo, suggesting that ZEB1 binds to the *CD34* promoter (Fig. 5d) and likely regulates its expression. In addition, we found that other vascularization related factors such as VEGFR1, VEGFR2, VE-cadherin and CD105 were down-regulated when ZEB1 was knocked down. We also performed an EMSA on *CD105*, an important angiogenic factor for tumor growth [32], but could not detect a ZEB1 binding complex (Supplemental Fig. S2a), suggesting that ZEB1 may not directly bind to regulate *CD105*. VE-cadherin, also known as CDH5, is an indicator for vascular mimicry [25]. Using a ChIP assay, we found that ZEB1 binds to the promoter of *CDH5*, (Fig. 5e) and, thus, may participate in vasculogenic mimicry through *CDH*5 regulation. We also found that in case of *PECAM1*, a mature vessel marker, its expression was not affected by ZEB1 knockdown and its promoter did not bind to ZEB1 (Supplemental Fig. S2b), indicating that ZEB1 may not affect vessel maturation. How *VEGFR1, VEGFR2* and *CD105* are regulated by ZEB1 remains unclear and needs further investigation.

## Discussion

**Fig. 6:**
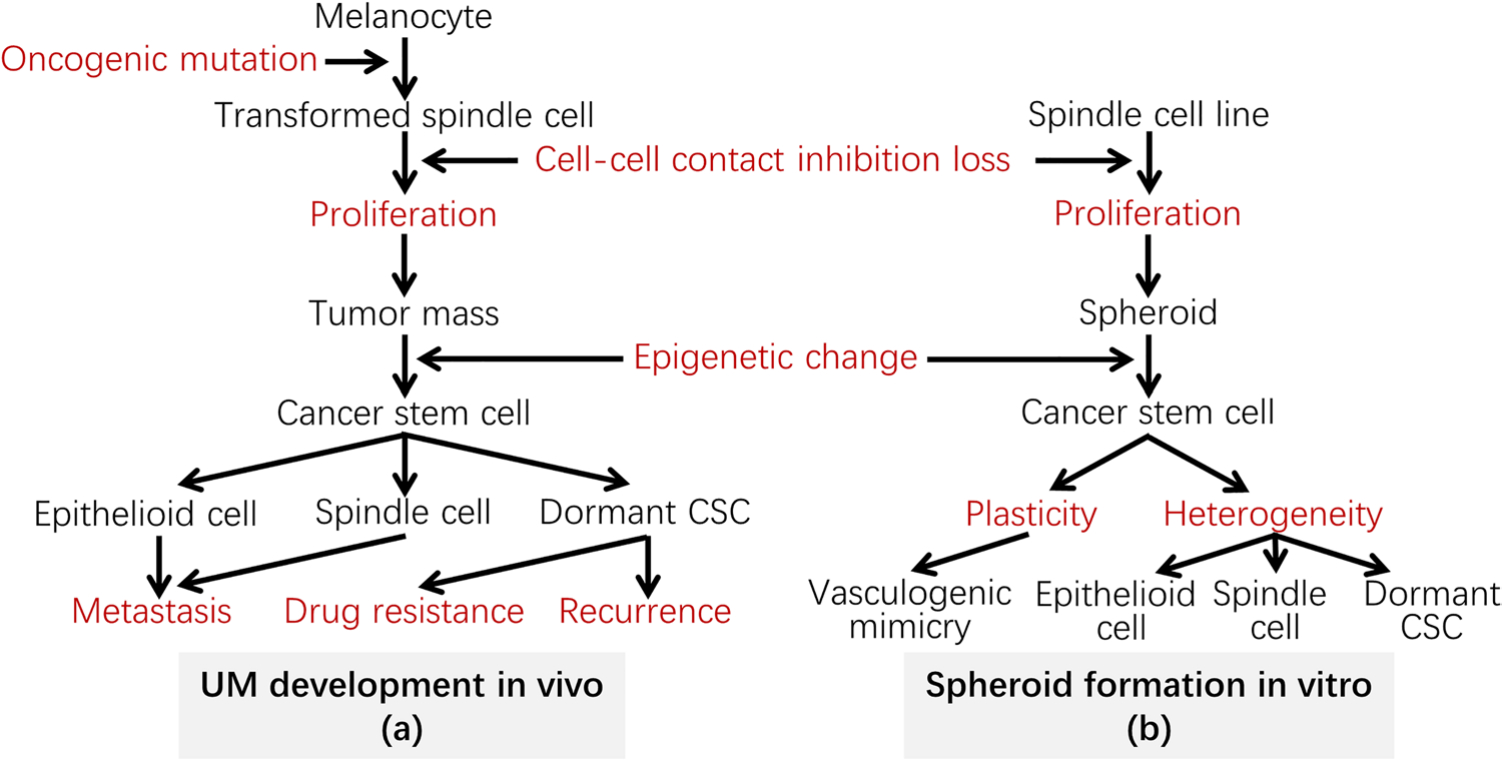
Schematic diagrams of hypothetic development and progression of UM in vivo (**a**) and in the UM cellular spheroid model to mimic such processes in vitro (**b**). CSC, cancer stem cell

ZEB1 is known to play pleiotropic roles in cancer, including promoting EMT by repression of CDH1 to make tumor cells mobile and invasive, and by enhancing cell proliferation by repression of cyclin-dependent kinase (CDK) inhibitor genes such as P15 and P21 [33]. As reported before, the expression levels of ZEB1 in UM cells are positively related to their proliferative ability. ZEB1^high^ C918-derived tumors were found to grow faster and to be more invasive than ZEB1^low^ OCM1-derived tumors, and ZEB1-overexpressing OCM1 (OCM1-ZEB1) cell-derived tumors grew faster and bigger with more Ki67 positive cells than OCM1-vector-derived tumors [21]. In addition, ZEB1 has been shown to participate in the development of cancer stem cells [34, 35] which can remain dormant with drug-resistant properties and can be reactivated under certain microenvironmental stimuli to differentiate into various cell types to endow tumor heterogeneity, to produce more invasive tumor cells to penetrate adjacent healthy tissues, and to survive long in circulation to reach remote areas for metastasis. There is a long debate on whether cancer stem cells originate from normal stem cells or from transformed cells [36]. Except for blood cells, normal differentiated cells possess cell–cell contact inhibition that prevents them from proliferation under normal conditions. But, transformed tumor cells have lost cell–cell contact inhibition properties and continue to proliferate, resulting in 3D solid tumor formation. We hypothesize that solid tumor formation in vivo may lead to various microenvironmental conditions, including hypoxia, that may reactivate essential stemness genes such as MYC and HIF1A to reprogram tumor cells into stem-like cells (Fig. 6). This in vivo tumor formation process can be recapitulated by tumor cell spheroid formation in a culture which is supposed to propel epigenetic reconstruction of the cells within the spheroid and leads to the acquisition of stem cell like properties (Fig. 6) [37]. Hypoxia and detachment from the extracellular matrix activate a stress-induced apoptosis program in most cancer cells [28], but not in stress insensitive cancer stem cells. Hence cancer stem cells thrive after spheroid formation.

UM cancer cells are heterogeneous and it has been reported that some UM cell lines, like OMM2.5, contain stem-like cells [38]. There are no authoritative data to estimate the occurrence of histological UM cell types. Based on some published UM cases, the percentages of the three major cellular phenotypes vary widely, and it appeared that the mixed cell types were predominant among UMs [3, 39–41]. The OMM2.5 cell line can form in vitro both large holoclone-like clones, consisting of tightly packed spindle shaped cells, and small paraclone-like clones, consisting of flat polygonal cells and some dendritic cells [38]. Interestingly, it was found that cells isolated from individual holoclone-like clones could produce both holoclone and paraclone types while cells isolated from paraclone-like clones gave only rise to paraclone-like cells [38]. Based on these observations, it appears that OMM2.5 holoclone-like cells are similar to OCM1 spindle cells with stem-like properties, whereas OMM2.5 paraclone-like cells more resemble C918 epithelioid cells with highly differentiated cancer cell properties.

Although there are some reservations as to the extent to which spheroid formation reflects the cancer stem cell (CSC) phenotype, the capability to form spheroids in vitro is regarded as a convenient surrogate to evaluate the functionality of CSCs because of the propensity of stem cells to propagate as spheroid bodies [42, 43]. Thus, tumor-derived spheroid cultures have been regarded as one of the criteria for CSCs [9]. We identified a subpopulation negative for Hoechst dye in the UM cell line OCM1 after spheroid formation in culture. These cells were morphologically different from their parental OCM1 cells and capable of remote migration. They also possessed stem cell-like properties such as ABC transporters on the cellular membrane that can pump out small chemicals and result in multidrug resistance [44]. Cancer stem cells, also known as “side population”, play an important role in cancer metastasis since they can develop tumors in remote areas [45]. We found that a small percentage of spheroid-derived OCM1 cells gained stem cell like properties after spheroid formation, which is in agreement with a previous report [37], and suggests that UM cell spheroid cultures can reprogram tumor cells into cells expressing stem cell markers including MYC, TERT, CD44, BMI1, ABCG2 and ABCB1, among which MYC is one of the four essential factors that can reprogram fibroblasts into so-called induced pluripotent stem cells (iPSC) similar to embryonic stem cells (ESC) [46] while activation of human TERT can transform primary cultured cells with a limited passaging capacity into immortalized cells [47]. Since we found that OCM1 spheroid cultures can generate pigmented epithelioid cells, we believe that it recapitulates in vivo tumor progression. We hypothesize that in the early stages of tumorigenesis, transformed UM cells mostly maintain their spindle phenotype, and that only a small percentage of cells become cancer stem cells in later stages of tumor progression when the primary tumor increases in size and generate cells with different morphologies. Both spindle and epithelioid cells in UMs may result from such a side population in vivo (Fig. 6). We found that morphologically, 3D tumor-derived epithelioid cells, as with those generated by side populations in vitro, could migrate through the bloodstream in grafted animals and metastasize to the liver. In later stage of mixed UMs, rapidly growing epithelioid cells with a high ZEB1 expression [21] may eventually become predominant, taking over spindle cells with a low ZEB1 expression [21] due to their slow proliferation rates. This hypothesis was partly confirmed by our current study in which xenograft OCM1 tumors showed a mixture of a large proportion of epithelioid cells and a small proportion of spindle cells, whereas C918 only led to epithelioid tumors. Moreover, downregulation of ZEB1 may reduce the UM cell proliferation rate and tumor size and, thereby, delay the generation of cancer stem cells [21]. In addition, ZEB1 may directly bind and thereby regulate the expression of multiple cancer stem cell markers including MYC and ABCB1. Hence, ZEB1 may be an important factor in the generation of UM cancer stem cells.

Vasculogenic mimicry, regarded as a sign of cancer cell plasticity, was initially found in UM and to be related to a poor prognosis [48]. Cancer cells under 3D culture conditions were more likely to develop chemo- and radio-resistance [49]. Invasive epithelioid C918 cells showed vasculogenic mimicry in 3D culture whereas low invasive spindle OCM1 cells only formed spheroids [22]. A high expression of ZEB1 in C918 cells may contribute to such vasculogenic mimicry. Folberg et al. observed co-expression of UM markers and the vessel precursor CD34 [50], but we found that the mature endothelial marker CD31 did not show such a co-expression (unpublished data), indicating the plasticity of UM cells capable of expressing vessel precursors and developing neovascularized structures. We found that CD34 and ZEB1 in patient-derived intraocular UM adjacent sections were present at the same locations, indicating that ZEB1 expression may relate to vasculogenic mimicry. Furthermore, we found that ZEB1 can bind to the upstream promoter of the vasculogenic mimicry marker *CDH5* and propel a string of vasculogenic factors such as VEGFR1 and VEGFR2. In addition, we found by EMSA that ZEB1 is bound to the *CD34* sequence in living cells. These data support our presupposition that ZEB1 facilitates vasculogenic mimicry in UM.

Although therapeutic methods to reduce ZEB1 expression are limited, RNA interference, particularly through the ZEB1 suppressor miR-200 family, have been considered for potential therapeutic use [51]. As yet, no direct ZEB1 inhibitor has been identified, although the indirect small molecule NSC95397 has been found to hamper the interaction of ZEB1 with its partner CtBP for the repression of target genes [52, 53]. Previously, we found that inactivation of ZEB1 by the ZEB1-CtBP inhibitor NSC95397 in mouse vascular endothelial cells significantly reduced their proliferation, and that topical application of NSC95397 to alkali-treated mouse corneas relieved ZEB1-associated corneal neovascularization [54]. These results suggest that NSC95397 may have the potential to treat UM both locally by direct delivery to the vitreous when it is a small tumor, or systematically by intravenous injection when it is a large tumor with a high potential to metastasize.

## Conclusions

Our data indicate that a subpopulation of UM cells exhibits stem cell like properties, giving rise to UM heterogeneity and plasticity. Spheroid formation in culture recapitulates UM growth in vivo and, thereby, may facilitate drug development. ZEB1 appears to play a key role in the development of UM cancer stem cell properties and vasculogenic mimicry.

## Supplementary Information

Below is the link to the electronic supplementary material.

Supplementary file1 (DOCX 1907 kb)

## Data Availability

All data are available within the article and supplementary files, or from the authors upon request.
