# Supplementary material for: Spheroid-induced heterogeneity and plasticity of uveal melanoma cells
Source: Cell Oncol (Dordr). 2022 Apr 11;45(2):309–21. doi: 10.1007/s13402-022-00671-y (PMC9050762; doi:10.1007/s13402-022-00671-y)
Supplement: Supplementary file 1 — Supplementary file1 (DOCX 1907 kb) [file 13402_2022_671_MOESM1_ESM.docx]

**Supplemental Table S1.** Primers used for PCR (Pro, Promoter)

|  | | |  |
| --- | --- | --- | --- |
| **Primer name** | **Sequence (5'—3')** | **Tm ˚C** | **Amplicon (bp)** |
| Hs ZEB1 LP | CTACAACAACAAGACACTGCTGT | 55.4 | 176 |
| Hs ZEB1 RP | TGTTCTTTCAGAGAGGTAAAGCG | 54.8 |  |
| Hs MYC LP | CCTACCCTCTCAACGACAGC | 57.3 | 248 |
| Hs MYC RP | CTCTGACCTTTTGCCAGGAG | 55.6 |  |
| Hs MYC Pro LP | CAGTGCGTTCTCGGTGTG | 56.4 | 127 |
| Hs MYC Pro RP | CCTCTCAAACCCTCTCCCTT | 56.1 |  |
| Hs TERT LP | AACCTTCCTCAGCTATGCCC | 57 | 210 |
| Hs TERT RP | GCGTGAAACCTGTACGCCT | 58 |  |
| Hs TERT Pro LP | GATTCGACCTCTCTCCGCT | 56.3 | 229 |
| Hs TERT Pro RP | AAGGTGAAGGGGCAGGAC | 57.4 |  |
| Hs CD44 LP | GCAAGGCTTTCAATAGCACC | 54.6 | 146 |
| Hs CD44 RP | GTTGTTTGCTGCACAGATGG | 55 |  |
| Hs CD44 Pro LP | CCCTATGACAGGCCATCAGT | 56.5 | 138 |
| Hs CD44 Pro RP | GGGAGTTGGTGAATCTTCCA | 54.5 |  |
| Hs BMI1 LP | ACCTGGAGAAGGAATGGTCC | 56.4 | 191 |
| Hs BMI1 RP | GTACTGGGGCTAGGCAAACA | 57.1 |  |
| Hs ABCB1 LP | GCGACAGGAGATAGGCTGGTT | 59 | 220 |
| Hs ABCB1 RP | AGCTGACAGTCCAAGAACAGGA | 58.1 |  |
| Hs ABCB1 Pro LP | TAGTGCTTTCAAGCCTGCCT | 56.8 | 214 |
| Hs ABCB1 Pro RP | TCTGGTTGCTTCCTGAAGTG | 54.9 |  |
| Hs ABCG2 LP | CAGTTCTCAGCAGCTCTTCGG | 57.9 | 225 |
| Hs ABCG2 RP | GCCAGTTGTAGGCTCATCCAAG | 58 |  |
| Hs PECAM1 LP | TCCGGATCTATGACTCAGGG | 55.4 | 161 |
| Hs PECAM1 RP | ACAGTTGACCCTCACGATCC | 56.8 |  |
| Hs CD105 LP | TGTCTCACTTCATGCCTCCAGCT | 60.3 | 378 |
| Hs CD105 RP | AGGCTGTCCATGTTGAGGCAGT | 61.1 |  |
| Hs VEGFR1 LP | GACCTGGAGTTACCCTGATGAAA | 56.3 | 76 |
| Hs VEGFR1 RP | GGCATGGGAATTGCTTTGG | 55 |  |
| Hs VEGFR2 LP | TCAGGCAGCTCACAGTCCTAGA | 59.5 | 71 |
| Hs VEGFR2 RP | ACTTGTCGTCTGATTCTCCAGGTT | 57.9 |  |
| Hs CDH5 LP | ACAAGGACACTGGCGAAAAC | 54.7 | 218 |
| Hs CDH5 RP | CCCCTTCAGGATTTGGTACA | 56.9 |  |
| Hs CDH5 Pro LP | TGGACAAGCACCTTAAACCC | 55.3 | 163 |
| Hs CDH5 Pro RP | CAGCTCTGGGACTCTGAACC | 57.3 |  |
| Hs CD34 LP | CACCCTGTGTCTCAACATGG | 55.8 | 213 |
| Hs CD34 RP | AACATTTCCAGGTGACAGGC | 55.6 |  |
| Hs ACTB LP | GGACTTCGAGCAAGAGATGG | 55.3 | 234 |
| Hs ACTB RP | AGCACTGTGTTGGCGTACAG | 57.9 |  |

**Supplemental Fig. S1.** Adherent monolayer cultured C918 and OCM1 cells are all Hoechst dye positive.

**
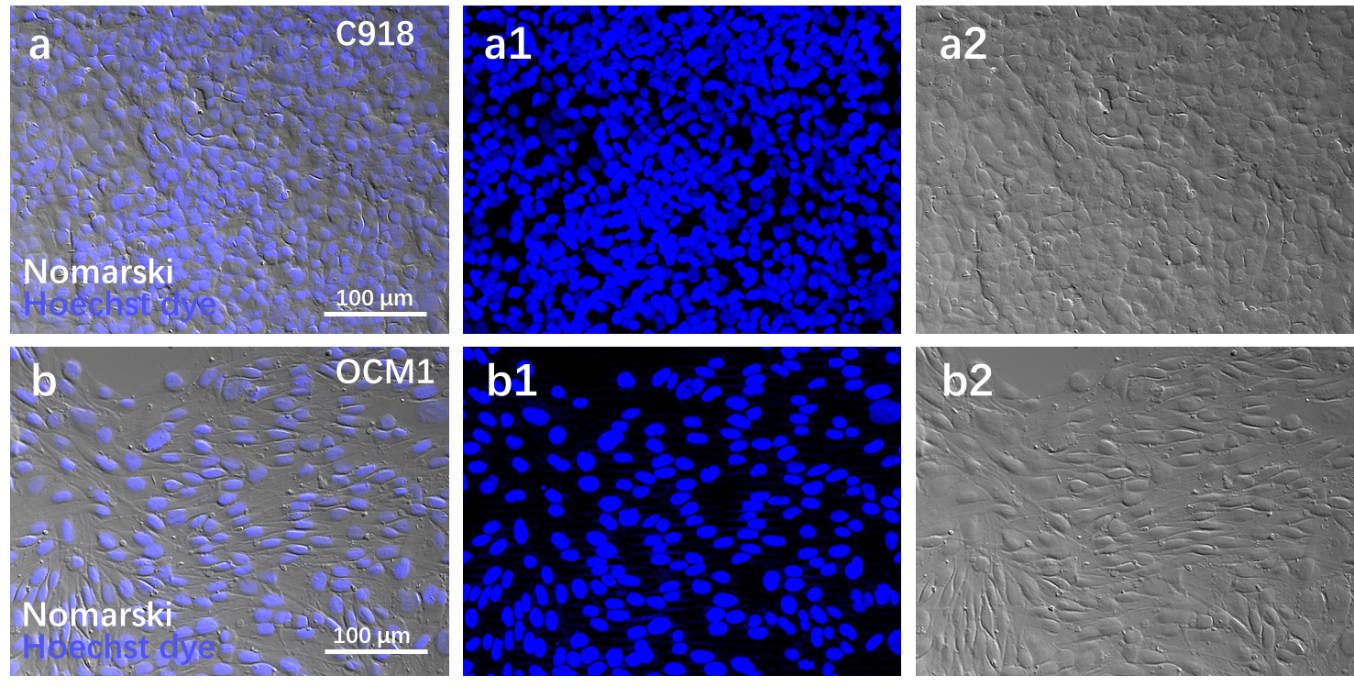
**

**Supplemental Fig. S2. (a)** ZEB1 is not detected by EMSA to bind to the promoter sequence of the CD105 gene. **(b)** ZEB1 is also not detected by ChIP assay to bind to the promoter sequence of the PECAM1 gene.

**
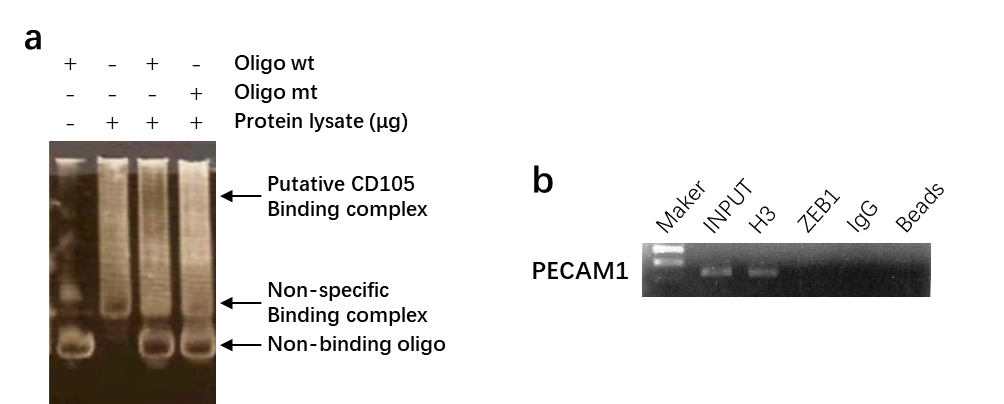
**
